# Supplementary material for: Assessment of research ethics education offerings of pharmacy master programs in an Arab nation relative to top programs worldwide: A qualitative content analysis
Source: PLoS One. 2021 Feb 19;16(2):e0238755. doi: 10.1371/journal.pone.0238755 (PMC7895361; doi:10.1371/journal.pone.0238755)
Supplement: S1 Table — Listed are universities that offer master programs with mixed coursework and research requirements. (PDF) [file pone.0238755.s001.pdf]

S1 Table. Research ethics education offerings of pharmacy master programs in Jordan. Listed are universities that offer master programs with mixed coursework and research requirements.

University name	Name of master program in Pharmacy	Name of course offering research ethics education	Is it a core course or an elective course?	Course description	Is the research ethics instruction offered the only focus of the course (i.e. a dedicated research ethics course) or an imbedded material (i.e. one component of the course)?	Keywords from the course description	Course contents related to research ethics
Jordan University of Science and Technology	Clinical Pharmacy	Research Methodology	Core course	The course involves the study of the different aspects of scientific research including data analysis and problems that may face the researcher. The course also introduces research methodologies, the application of research approaches to health professions, and the statistical techniques used in comparing researches results and ethics.	Imbedded research ethics instruction.	Problems, research approaches, ethics	1) Research involving animals: animal handling, euthanasia.<br>2) Protecting human research participants:<br>a) History: Goals and Principles of Human Subjects Protection, Timeline of Important Historical Events (Nazi Medical War Crimes, The Nuremburg Code, Syphilis Study at Tuskegee).<br>b) Codes and regulations: The Belmont Report – Ethical Principles and Guidelines for the Protection of Human Subjects of Research, HHS Regulations for the Protection of Human Subjects, 45 CFR 46.<br>c) Respect for Persons: informed consent process (fundamental aspects and elements), requirements for documentations, waivers, diminished autonomy, legally authorized representatives, participation of pregnant women, assent from children and permission from parents, obtaining informed consent from prisoners, community consent, emergency research.<br>d) Beneficence: risks and benefits, privacy and confidentiality, IRB (definition, role, membership, expedited IRB review), Data and safety monitoring, compensation for research participants, undue influence, therapeutic misconception, equipoise.<br>e) Justice: Individual Justice and Social Justice , Equity vs. Equality in Human Subjects Research, distribution of the benefits and burdens of research, NIH Inclusion Policies: Women and Minorities, Inclusion of Children in Research, Justice and the Use of Placebos, Incomplete Disclosure and Deception, Waiver of Informed Consent, To Debrief or Not to Debrief, Fairness in International Research, Standards and Assurances for International Research, IRB Review for Research in International Settings, Local Cultural Norms and Informed Consent.
Jordan University of Science and Technology	Clinical Pharmacy	Design of Clinical Trials	Elective course	The course deals with various clinical trial designs and carefully considers the medical, ethical and therapeutic requirements. Since there are various designs, the course also deals with the statistical methods used in the analysis and interpretation of results emerging from such clinical trials.	Imbedded research ethics instruction.	Ethical Requirements	No course content available. The course is listed in the program’s syllabus, however it has not been offered for the last 9 years at least.

Jordan University of Science and Technology	Jordan University of Science and Technology	Jordan University of Science and Technology
Medicinal Chemistry and Pharmacognosy	Pharmaceutical Technology	Clinical Pharmacy
Research Methodology	Research Methodology	Design of Clinical Surveys
Core course	Core course	Elective course
This course provides students with knowledge and skills required to conduct research in a scientific approach. Practical application of different aspects and special problems encountered in research is also discussed.	Practical application of different aspects and special problems encountered in research.	The course includes topics on clinical survey design requirements such as clarity and integrity. It also reviews the common errors in the design of surveys and the appropriate ways of avoiding them. The ethical, medical and social considerations as integral parts of each survey are also discussed.
Imbed research ethics instruction.	Imbedded research ethics instruction.	Imbedded research ethics instruction.
Special problems	Special problems	Ethical consideration
1) Research involving animals: animal handling, euthanasia.<br>2) Protecting human research participants:<br>a) History: Goals and Principles of Human Subjects Protection, Timeline of Important Historical Events (Nazi Medical War Crimes, The Nuremburg Code, Syphilis Study at Tuskegee).<br>b) Codes and regulations: The Belmont Report – Ethical Principles and Guidelines for the Protection of Human<br>Subjects of Research, HHS Regulations for the Protection of Human Subjects, 45 CFR 46.<br>c) Respect for Persons: informed consent process (fundamental aspects and elements), requirements for documentations, waivers, diminished autonomy, legally authorized representatives, participation of pregnant women, assent from children and permission from parents, obtaining informed consent from prisoners, community consent, emergency research.<br>d) Beneficence: risks and benefits, privacy and confidentiality, IRB (definition, role, membership, expedited IRB review), Data and safety monitoring, compensation for research participants, undue influence, therapeutic misconception, equipoise.<br>e) Justice: Individual Justice and Social Justice , Equity vs. Equality in Human Subjects Research, distribution of the benefits and burdens of research, NIH Inclusion Policies: Women and Minorities, Inclusion of Children in Research, Justice and the Use of Placebos, Incomplete Disclosure and Deception, Waiver of Informed Consent, To Debrief or Not to Debrief, Fairness in International Research, Standards and Assurances for International Research, IRB Review for Research in International Settings, Local Cultural Norms and Informed Consent.	1) Research involving animals: animal handling, euthanasia. 2) Protecting human research participants: a) History: Goals and Principles of Human Subjects Protection, Timeline of Important Historical Events (Nazi Medical War Crimes, The Nuremburg Code, Syphilis Study at Tuskegee).b) Codes and regulations: The Belmont Report – Ethical Principles and Guidelines for the Protection of HumanSubjects of Research, HHS Regulations for the Protection of Human Subjects, 45 CFR 46.c) Respect for Persons: informed consent process (fundamental aspects and elements), requirements for documentations, waivers, diminished autonomy, legally authorized representatives, participation of pregnant women, assent from children and permission from parents, obtaining informed consent from prisoners, community consent, emergency research.d) Beneficence: risks and benefits, privacy and confidentiality, IRB (definition, role, membership, expedited IRB review), Data and safety monitoring, compensation for research participants, undue influence, therapeutic misconception, equipoise.e) Justice: Individual Justice and Social Justice , Equity vs. Equality in Human Subjects Research, distribution of the benefits and burdens of research, NIH Inclusion Policies: Women and Minorities, Inclusion of Children in Research, Justice and the Use of Placebos, Incomplete Disclosure and Deception, Waiver of Informed Consent, To Debrief or Not to Debrief, Fairness in International Research, Standards and Assurances for International Research, IRB Review for Research in International Settings, Local Cultural Norms and Informed Consent.	No course content available. The course is listed in the program's syllabus, however it has not been offered for the last 9 years at least.

University of Petra	Amman Al-Ahlya University	Al-Zaytoonah University	Applied Science University	Al-Isra University	University of Jordan	University of Jordan
Pharmaceutical Sciences	Pharmaceutical Sciences	Pharmaceutical Sciences	Pharmaceutical Sciences	Pharmacy	Pharmaceutical Sciences	Clinical Pharmacy
None	None	None	None	None	None	Clinical Research Methods and Statistics
-	-	-	-	-	-	Core course
-	-	-	-	-	-	This course introduces the students to the fundamentals of research including measurement, reliability, validity, ethical concerns, clinical research design; data analysis and presentation.
No-	No-	No-	No-	No-	No-	Imbedded research ethics instruction.
-	-	-	-	-	-	Ethical Concerns
-	-	-	-	-	-	a) Ethical principles of research: Honesty, Objectivity, Integrity, Carefulness, Openness, Respect for Intellectual Property, Confidentiality, Responsible Publication, Responsible Mentoring, Social Responsibility, Non-Discrimination, Competence, Human Subjects Protection.<br>b) Examples of Research Misconduct: The Nuremberg Code of 1946, Declaration of Helsinki, Tuskegee Syphilis Study 1932-1972, and Belmont report.<br>c) Introducing the informed consent: Definition and elements.<br>d) Introducing the IRB: Definition of the IRB and its role, Jordan Law, members of the IRB, Which studies need IRB, Purpose and use of IRB, Objectives of IRB, responsibilities of IRB, The IRB review process, IRB continuing review of a trial, IRB and informed consent, IRB and payment to subjects, Independent IRB, voting in the IRB, IRB and protocol amendments.<br>e) Reporting research/Scientific publication: Authorship credit, Scientific misconduct, Ethical violation (duplicate publication, conflict of interest, misinterpretation of findings, confidential data, bypassing peer review).
