# Supplementary material for: Assessment of research ethics education offerings of pharmacy master programs in an Arab nation relative to top programs worldwide: A qualitative content analysis
Source: PLoS One. 2021 Feb 19;16(2):e0238755. doi: 10.1371/journal.pone.0238755 (PMC7895361; doi:10.1371/journal.pone.0238755)
Supplement: S2 Table — Listed are all top 10 universities and all pharmacy master programs they offer by mixed coursework and research requirements. (PDF) [file pone.0238755.s002.pdf]

S2 Table. Research ethics education offerings of pharmacy master programs in the top 10 universities globally by subject of pharmacy and pharmacology. Listed are all top 10 universities and all pharmacy master programs they offer by mixed coursework and research requirements.

University Name and rank	Name of master program in pharmacy	Name of pharmacy School /department	Name of course offering research ethics instruction	Is it a core course or an elective course?	Is the research ethics instruction offered the only focus of the course or an imbedded material?	Course's/module's description	Key words	Notes	Program's/course's website
University of Harvard (1st)	No MSc mixed (taught) program is offered.	University of Harvard does not have a Pharmacy School.	-	-	-	-	-	Note from QS: "While the university doesn't have a pharmacy department, pharmacology features in many of the courses offered at Harvard Medical School."	N/A
University of Monash (2nd)	Master of Clinical Pharmacy	Pharmacy and Pharmaceutical Sciences	PGC5107 - Introduction to clinical research	Core module	Imbedded research ethics instruction.	This unit will enable students to develop their skills in performing practice based research. Content will cover topics such as: performing practice based research, writing project/grant proposals and ethics submissions, qualitative versus quantitative research, managing and analyzing data.	Ethics submissions	-	https://monash.edu/pubs/2017handbooks/units/PGC5107.html
University of Monash (2nd)	Master of Clinical Pharmacy	Pharmacy and Pharmaceutical Sciences	Clinical research 2	Elective module	Imbedded research ethics instruction.	This unit will enable students to consolidate their skills in performing practice based research. There will be limited didactic lectures with the majority of time allocated to collection of data consistent with the methodology developed. One of the course outcomes is to demonstrate understanding of research ethics as encountered at their practice site and at Monash University by gaining appropriate ethics approval.	Research Ethics, ethics approval	-	https://monash.edu/pubs/2017handbooks/units/PGC5108.html

University College London (8th)	University of Cambridge (3rd)	University of Oxford (4th)
MSc in Medicinal Natural Products and Phytochemistry	No MSc mixed (taught) program is offered by the department of pharmacology.	MSc in Pharmacology
School of Pharmacy	Department of Pharmacology	Medical Sciences Division, Department of Pharmacology
N/A	-	N/A
-	-	-
No enough information is available for the program in the school's website.	-	Description of modules is not available online
-	-	-
-	-	-
No enough information is available for the program in the school's website.	The department of pharmacology only offers MPhil Pharmacology program, which is a master program entirely by research.	No key words related to research ethics are found online in the program's aims, features, and structure.
http://www.ucl.ac.uk/pharmacy/courses-and-phd/msc-mednatprod	http://www.graduate.study.cam.ac.uk/courses/departments/blph	https://pharm.ox.ac.uk/study-with-us/msc-taught-course-in-pharmacology

University College London (8th)	University College London (8th)	University College London (8th)
MSc in Drug Discovery and Development	MSc in Drug Discovery and Development	MSc in Pharmaceutics
School of Pharmacy	School of Pharmacy	School of Pharmacy
Pharma management 1	The process of drug development (TPODD2)	Personalised medicine
Core module	Core module	Core module
Imbedded research ethics instruction.	Imbedded research ethics instruction.	Imbedded research ethics instruction.
This module will cover the following key areas:<br>1) The team approach to Research and Clinical Development. The module will discuss the important input from: • Clinical Development • Intellectual property. • Commercial Teams.<br>2) Global Regulatory Affairs and the different steps involved:<br>• Regulatory Objectives<br>• Regulatory Agencies.<br>• Global Regulatory Package.<br>• Global Process<br>• Hearings and approvals<br>• What does an approval provide<br>• Risk Management and post approval obligations.<br>3) Clinical Development<br>• Study types, Phase 1 2 and 3<br>• The requirements pre tox for commencement of each phase<br>• Multi-disciplinary teams. The role the scientist may play in metabolite identification, sample analysis etc.<br>• Centre selection<br>• Advisory board, Safety Board<br>• Regulatory clearance<br>• Ethics boards<br>• Use of contract organisations<br>• Data interpretation, audits	Clinical Development: The choice, preparation for and execution of the four phases of clinical trials are investigated. External experts from Celgene, CRUK, plus Quintiles and the NHS discuss a variety of issues and expertise.	This module will provide a perspective in pharmacogenomics applied to clinical and pharmaceutical research, with particular emphasis on patient-centric medicine. The focus of the module will be on the development and use of personalized medicine for the treatment of diseases. The module will also provide an introduction to the use of personalized medicine for prevention and diagnosis of diseases. Students will develop a theoretical knowledge and understanding of personalized medicine in pharmaceutical sciences alongside drug discovery, clinical pharmacology, pharmacogenetics, pharmacokinetics/pharmacodynamics, tailored drug delivery, epidemiology, ethics and governance of individualized therapies
team approach, regulatory, risk management approvals, obligations, safety board, ethics board	issues, expertise	ethics
-	-	-
http://www.ucl.ac.uk/pharmacy/courses-and-phd/msc-drug-discovery-develop	http://www.ucl.ac.uk/pharmacy/courses-and-phd/msc-drug-discovery-develop	http://www.ucl.ac.uk/pharmacy/courses-and-phd/msc-pharmaceutics

University College London (8th)	University College London (8th)
MSc in Drug Discovery and Pharma Management	MSc in Drug Discovery and Pharma Management
School of Pharmacy	School of Pharmacy
Pharma management 1	The process of drug development (TPODD2)
Core module	Core module
Imbedded research ethics instruction.	Imbedded research ethics instruction.
This module will cover the following key areas:<br>1) The team approach to Research and Clinical Development. The module will discuss the important input from: • Clinical Development • Intellectual property. • Commercial Teams.<br>2) Global Regulatory Affairs and the different steps involved:<br>• Regulatory Objectives<br>• Regulatory Agencies.<br>• Global Regulatory Package.<br>• Global Process<br>• Hearings and approvals<br>• What does an approval provide<br>• Risk Management and post approval obligations.<br>3) Clinical Development<br>• Study types, Phase 1 2 and 3<br>• The requirements pre tox for commencement of each phase<br>• Multi-disciplinary teams. The role the scientist may play in metabolite identification, sample analysis etc.<br>• Centre selection<br>• Advisory board, Safety Board<br>• Regulatory clearance<br>• Ethics boards<br>• Use of contract organisations<br>• Data interpretation, audits	Clinical Development: The choice, preparation for and execution of the four phases of clinical trials are investigated.<br>External experts from Celgene, CRUK, plus Quintiles and the NHS discuss a variety of issues and expertise.
team approach, regulatory, risk management approvals, obligations, safety board, ethics board	issues, expertise
-	-
http://www.ucl.ac.uk/pharmacy/courses-and-phd/msc-drug-discovery-develop	http://www.ucl.ac.uk/pharmacy/courses-and-phd/msc-drug-discovery-develop

King's College London (7th)	University College London (8th)	University College London (8th)	University College London (8th)
Msc in Clinical Pharmacology	Master of Research (MRes) in Drug Sciences	MSc in Clinical Pharmacy, International Practice and Policy	MSc in Pharmaceutical Formulation and Entrepreneurship
Faculty of Life Sciences and Medicine, Department of Pharmacy, Pharmacology, and Forensic Sciences	School of Pharmacy	School of Pharmacy	School of Pharmacy
Advanced clinical pharmacology	N/A	N/A	Personalised medicine
Core module	-	-	Elective module
Imbedded research ethics instruction.	Description of individual modules is not available online.	Description of modules is not available online	Imbedded research ethics instruction.
Over four days this module provides advanced training in the principles of clinical pharmacology essential to drug development. This includes practical and ethical issues associated with non-patient volunteer studies, the design of such studies and interpretation of the results. Completion of this module will enable you to take an active role in a multidisciplinary team to design and analyze clinical pharmacology studies.<br>Key topics include: Non-patient volunteer studies, planning a clinical development strategy, use of pharmacodynamic and pharmacokinetic measures in clinical pharmacology studies.<br>You will take part in practical sessions to plan a clinical development strategy for a potential new medicine, prepare a study protocol, analyze study results and learn how to present clinical pharmacology study results through both verbal and written presentations.	-	-	This module will provide a perspective in pharmacogenomics applied to clinical and pharmaceutical research, with particular emphasis on patient-centric medicine. The focus of the module will be on the development and use of personalized medicine for the treatment of diseases. The module will also provide an introduction to the use of personalized medicine for prevention and diagnosis of diseases. Students will develop a theoretical knowledge and understanding of personalized medicine in pharmaceutical sciences alongside drug discovery, clinical pharmacology, pharmacogenetics, pharmacokinetics/pharmacodynamics, tailored drug delivery, epidemiology, ethics and governance of individualized therapies
Ethical issues, multidisciplinary team	-	-	ethics
-	No enough information to assess research ethics instruction offerings by the program.	No enough information to assess research ethics instruction offerings by the program.	-
https://www.kcl.ac.uk/study/courses-data/modules/7/Advanced-Clinical-Pharmacology-7BBM0006.aspx	http://www.ucl.ac.uk/pharmacy/courses-and-phd/mres-drug-sciences	http://www.ucl.ac.uk/pharmacy/courses-and-phd/msc-clinical-pharmacy	http://www.ucl.ac.uk/pharmacy/courses-and-phd/msc-formulation-entrepreneurship

King's College London (7th)	King's College London (7th)	King's College London (7th)
MSc in Clinical Pharmacology	MSc in Clinical Pharmacology	MSc in Clinical Pharmacology
Faculty of Life Sciences and Medicine, Department of Pharmacy, Pharmacology, and Forensic Sciences	Faculty of Life Sciences and Medicine, Department of Pharmacy, Pharmacology, and Forensic Sciences	Faculty of Life Sciences and Medicine, Department of Pharmacy, Pharmacology, and Forensic Sciences
Drug development pharmacology	Clinical drug development	Biological and advanced therapies
Core module	Core module	Core module
Imbedded research ethics instruction.	Imbedded research ethics instruction.	Imbedded research ethics instruction.
This module covers the fundamental principles of pharmacology and how pharmacological concepts are applied in the context of pharmaceutical sciences. It will explore the role of pharmacology in the drug discovery/development process.<br>Key topics include: fundamental principles of pharmacology, such as dose-response relationship, benefit-risk ratio, agonism and antagonism, targets for drug action, in vitro techniques for assessing drug action, and pharmacokinetics/pharmacodynamics. There will be sessions on drugs and the cardiovascular system, drugs and blood, anti-infective drugs, drugs and cancer, and immunopharmacology	This module covers the development of a new medicine from early exploratory clinical development right through to the stage of applying for a license for marketing. We will highlight practical issues associated with running clinical trials, including large-scale multinational projects.<br>Key topics include: Clinical trial design, clinical end points, writing a protocol, project management, ethical and legal aspects of clinical research, logistics issues in clinical trials, clinical trials in children, data gathering for phase 2-4 studies, adverse events in clinical trials. You will take part in workshop sessions and practical exercises to "develop" a new molecule and learn how to apply the principles of statistics to clinical trial design.	This module provides advanced training in the area of medicines produced by biotechnology. Participants will study the new therapeutic technologies that are in development as well as the ethical and regulatory frameworks that apply to these products. The differences between the clinical development programmes required by these different technologies will also be explored. The areas covered include recombinant proteins, monoclonal antibodies, vaccines, both prophylactic and therapeutic, gene therapy, stem cells and epigenetics.
Benefit-risk ratio	Ethical aspects	Ethical frameworks, regulatory frameworks
-	-	-
https://www.kcl.ac.uk/study/courses-data/modules/7/Drug-Development-Pharmacology-7BBM0013.aspx	https://www.kcl.ac.uk/study/courses-data/modules/7/Clinical-Drug-Development-7BBP0005.aspx	https://www.kcl.ac.uk/study/courses-data/modules/7/Biological-and-Advanced-Therapies-7BBP0014.aspx

King's College London (7th)	King's College London (7th)	King's College London (7th)
MSc in Drug Development Science	MSc in Drug Development Science	MSc in Drug Development Science
Faculty of Life Sciences and Medicine, Department of Pharmacy, Pharmacology, and Forensic Sciences	Faculty of Life Sciences and Medicine, Department of Pharmacy, Pharmacology, and Forensic Sciences	Faculty of Life Sciences and Medicine, Department of Pharmacy, Pharmacology, and Forensic Sciences
Drug safety and ethics	Drug regulatory affairs	Advanced clinical pharmacology
Core module	Core module	Core module
Imbedded research ethics instruction.	Imbedded research ethics instruction.	Imbedded research ethics instruction.
In this module, two of the most important issues within the drug development process and the whole of clinical research are addressed. In addition to reviewing the general principles of medical ethics and how they apply to clinical research as defined within the various guidelines and statutes we will have presentations from patient groups and a member of an ethics committee. The issues around business ethics and marketing will also be explored.<br>We will look at the mechanisms of cellular toxicology, the value of spontaneous reporting and the use of large electronic data bases as means of evaluating the safety of medicines. We will look at safety issues from the point of view of the sponsor, the investigator and the regulator and learn how to develop and implement a risk management plan.	This module explores the principles that underpin drug regulation in the UK and worldwide. Over five days, we will consider the regulatory requirements for special patient populations, commercial issues from product life cycle development through to marketing, pharmacovigilance, and the handling of off label and unlicensed medicines.<br>Key topics include: European regulatory procedures, ethics committees, adverse drug reaction reporting, regulatory requirements in special populations (e.g, children, the elderly), advertising, internet sales, medical aspects of the Marketing Authorization, bioequivalence and generics, special licensing issues (such as for biologicals, devices, herbal and homoeopathic medicines), and pharmacovigilance (pre- and post-marketing).<br>You will take part in a series of workshops to learn about practical aspects of Clinical Trial Authorizations, preparation of Summaries of Product Characteristics and Patient information leaflets, risk minimization, and reclassification of the legal status of medicines.	Over four days this module provides advanced training in the principles of clinical pharmacology essential to drug development. This includes practical and ethical issues associated with non-patient volunteer studies, the design of such studies and interpretation of the results. Completion of this module will enable you to take an active role in a multidisciplinary team to design and analyze clinical pharmacology studies.<br>Key topics include: Non-patient volunteer studies, planning a clinical development strategy, use of pharmacodynamic and pharmacokinetic measures in clinical pharmacology studies.<br>You will take part in practical sessions to plan a clinical development strategy for a potential new medicine, prepare a study protocol, analyze study results and learn how to present clinical pharmacology study results through both verbal and written presentations.
Important issues, medical ethics, ethics committee, business ethics, safety issues, risk management	Ethical committees, regulatory requirements, risk minimization	Ethical issues, multidisciplinary team
-	-	-
https://www.kcl.ac.uk/study/courses-data/modules/7/Drug-Safety-and-Ethics-7BBP0011.aspx	https://www.kcl.ac.uk/study/courses-data/modules/7/Drug-Regulatory-Affairs-7BBP0007.aspx	https://www.kcl.ac.uk/study/courses-data/modules/7/Advanced-Clinical-Pharmacology-7BBM0006.aspx

King's College London (7th)	King's College London (7th)	King's College London (7th)
Msc in Pharmacology	MSc in Pharmacology	MSc in Drug Development Science
Faculty of Life Sciences and Medicine, Department of Pharmacy, Pharmacology, and Forensic Sciences	Faculty of Life Sciences and Medicine, Department of Pharmacy, Pharmacology, and Forensic Sciences	Faculty of Life Sciences and Medicine, Department of Pharmacy, Pharmacology, and Forensic Sciences
Experimental research project	Pharmacology of biological systems: practical skills	Clinical drug development
Core research module	Core module	Core module
Imbedded research ethics instruction.	Imbedded research ethics instruction.	Imbedded research ethics instruction.
The experimental project is carried out in the period from the beginning of April to the end of July (at least 14 weeks full-time in the laboratory) and is under the supervision of an individual member of staff. The aim is to provide students with a firm grounding in research techniques and to increase awareness of an up-to-date problem in pharmacology research. They will join an established research group and gain experience of a range of practical techniques. Assessment of the experimental project is by a poster presentation of results in July and a research report in the form of a scientific manuscript. The research supervisor also assigns a mark for laboratory skills, application and diligence.<br>The use of experimental animals, particularly transgenic rodents, is an important part of much of the pharmacological research at KCL, and in the wider research environment. UK law states that anyone wishing to work with live animals in the laboratory must hold a license from the Home Office. If the research project you select involves in vivo work, then you can attend a KCL training course for a Home Office license.	This module is designed to familiarize students with fundamentals of pharmacological experimentation and to encourage a hypothesis-led approach to research problems. The practical classes on this module are based around experiments in tissue and cellular pharmacology. Students will be required to use the data acquired during the practicals as the basis for writing three reports in the form of scientific papers. These reports will form the basis for assessment of the course unit.<br>The module also includes workshops on statistics, risk assessment and use of information resources.	This module covers the development of a new medicine from early exploratory clinical development right through to the stage of applying for a license for marketing. We will highlight practical issues associated with running clinical trials, including large-scale multinational projects. Key topics include: Clinical trial design, clinical end points, writing a protocol, project management, ethical and legal aspects of clinical research, logistics issues in clinical trials, clinical trials in children, data gathering for phase 2-4 studies, adverse events in clinical trials. You will take part in workshop sessions and practical exercises to “develop” a new molecule and learn how to apply the principles of statistics to clinical trial design.
Problems in pharmacology research	research problems, risk assessment	Ethical aspects
-	-	-
https://www.kcl.ac.uk/study/courses-data/modules/7/Experimental-Research-Project-MSc-7BBMM010.aspx	https://www.kcl.ac.uk/study/courses-data/modules/7/Pharmacology-Of-Biological-Systems-Practical-Skills-7bbmm014.aspx	https://www.kcl.ac.uk/study/courses-data/modules/7/Clinical-Drug-Development-7BBP0005.aspx

King's College London (7th)	King's College London (7th)	King's College London (7th)	King's College London (7th)
MSc in Pharmacy Practice	MSc in Pharmaceutical Technology	MSc in Pharmaceutical Analysis and Quality Control	MSc in Biopharmaceuticals
Faculty of Life Sciences and Medicine, Department of Pharmacy, Pharmacology, and Forensic Sciences	Faculty of Life Sciences and Medicine, Department of Pharmacy, Pharmacology, and Forensic Sciences	Faculty of Life Sciences and Medicine, Department of Pharmacy, Pharmacology, and Forensic Sciences	Faculty of Life Sciences and Medicine, Department of Pharmacy, Pharmacology, and Forensic Sciences
Could not find research ethics education offered in any of the courses	Could not find research ethics education offered in any of the courses	N/A	N/A
-	-	-	-
Neither	Neither	Description of modules is not available online	Description of modules is not available online
-	-	-	-
-	-	-	-
No research ethics instruction is offered by this program	No research ethics instruction is offered by this program	No enough information to assess research ethics instruction offerings by the program.	No enough information to assess research ethics instruction offerings by the program.
https://www.kcl.ac.uk/study/postgraduate/taught-courses/pharmacy-practice-prescribing-msc-pg-dip-pg-cert.aspx	https://www.kcl.ac.uk/study/postgraduate/taught-courses/pharmaceutical-technology-msc.aspx	https://www.kcl.ac.uk/study/postgraduate/taught-courses/pharmaceutical-analysis-and-quality-control-msc.aspx	https://www.kcl.ac.uk/study/postgraduate/taught-courses/biopharmaceuticals-msc.aspx

The University of Tokyo (9th)	University of Nottingham (6th)	University of California, San Francisco (5th)	Karolinska Institute (10th)
Master's degree in Pharmaceutical Sciences	Drug Discovery and Pharmaceutical Sciences MSc	Master of Science in Clinical Research Degree Program	Master courses in Pharmaceutical Medicine
Graduate School of Pharmaceutical Sciences	School of Pharmacy	School of Pharmacy	Executive and professional education
N/A	N/A	Responsible Conduct of Research (RCR)	Research ethics
-	-	Core module	Core module
Description of modules is not available online	Description of modules is not available online	Dedicated research ethics course	Dedicated research ethics course
-	-	Responsible Conduct of Research (RCR) helps people starting their research careers learn how to address the ethical issues that inevitably arise in research. The course addresses requirements and regulations for human-subjects research, including IRB approval and consent. Key topics include conflicts of interest, research misconduct, authorship, and ethical challenges related to research in resource-poor countries.	Research ethics, research methods, drug discovery and early development and pharmacometrics give a thorough grounding in the scientific development of a drug.
-	No key words related to research ethics are found in the overview of the course nor in the course details	RCR, ethical issues, regulations, IRB, consent, research misconduct, conflict of interest, authorship, ethical challenges	Research Ethics
No course description is provided in the website	No enough information to assess research ethics instruction offerings by the program.	Online course	Online course
http://www.u-tokyo.ac.jp/en/academics/grad_pharmaceutical.html	https://www.nottingham.ac.uk/pgstudy/courses/pharmacy/drug-discovery-and-pharmaceutical-sciences-msc.aspx	http://accelerate.ucsf.edu/training/responsible-conduct-research	http://ki.se/en/education/masters-courses-in-pharmaceutical-medicine-60-ects
